# The Impact of Nano-Hydroxyapatite Scaffold Enrichment on Bone Regeneration In Vivo—A Systematic Review

**DOI:** 10.3390/biomimetics9070386

**Published:** 2024-06-25

**Authors:** Dijana Mitić, Jelena Čarkić, Jelena Jaćimović, Miloš Lazarević, Milica Jakšić Karišik, Boško Toljić, Jelena Milašin

**Affiliations:** School of Dental Medicine, University of Belgrade, 11000 Belgrade, Serbia; jelena.carkic@stomf.bg.ac.rs (J.Č.); jelena.jacimovic@stomf.bg.ac.rs (J.J.); milos.lazarevic@stomf.bg.ac.rs (M.L.); milica.jaksic@stomf.bg.ac.rs (M.J.K.); bosko.toljic@stomf.bg.ac.rs (B.T.); jelena.milasin@stomf.bg.ac.rs (J.M.)

**Keywords:** nano-hydroxyapatite, bone scaffold, regeneration, bioactive components, tissue engineering

## Abstract

Objectives: In order to ensure improved and accelerated bone regeneration, nano-hydroxyapatite scaffolds are often enriched with different bioactive components to further accelerate and improve bone healing. In this review, we critically examined whether the enrichment of nHAp/polymer scaffolds with growth factors, hormones, polypeptides, microRNAs and exosomes improved new bone formation in vivo. Materials and Methods: Out of 2989 articles obtained from the literature search, 106 papers were read in full, and only 12 articles met the inclusion criteria for this review. Results: Several bioactive components were reported to stimulate accelerated bone regeneration in a variety of bone defect models, showing better results than bone grafting with nHAp scaffolds alone. Conclusions: The results indicated that composite materials based on nHAp are excellent candidates as bone substitutes, while nHAp scaffold enrichment further accelerates bone regeneration. The standardization of animal models should be provided in order to clearly define the most significant parameters of in vivo studies. Only in this way can the adequate comparison of findings from different in vivo studies be possible, further advancing our knowledge on bone regeneration and enabling its translation to clinical settings.

## 1. Introduction

While bone, as a complex and reactive tissue, does possess high renewal ability, the reconstruction of bone defects, especially critical-sized ones, still remains a tremendous therapeutic challenge. Although a single definition of what constitutes a critical-sized defect currently does not exist, it is generally regarded as one that will not heal spontaneously despite surgical stabilization and requires further treatment [1]. In this case, the therapeutic approach may include autologous or allogeneic bone grafting and synthetic bone substitutes [2]. Autologous grafts (tissue from the patient) are still regarded as the gold standard, and allografts (tissue harvested from another individual) as a favorable alternative. However, their use runs the risk of several associated drawbacks (additional surgery, limited bone supply, the possibility of infection, immune rejection, graft resorption, etc.) [3]. Therefore, development of various three-dimensional (3D) structural biomaterials (scaffolds) for bone repair purposes has taken center stage in recent years.

Bone scaffolds possess a porous microstructure which imitates the extracellular matrix (ECM) and ideally creates an appropriate regenerative microenvironment due to their biocompatibility [4], adequate mechanical properties, and biodegradability [5]. Among numerous biomaterials used for bone scaffolds, calcium phosphate-based ones, such as hydroxyapatite (HAp), are becoming increasingly attractive due to their chemical resemblance to the inorganic components of human bones and teeth [6]. HAp has a wide range of biomedical applications including bone scaffold implantation [7] and coating materials. It has also shown great promise as a drug carrier, either as doped hydroxyapatite for accelerated antibacterial properties [8], or as a sustained drug release mediator [9]. The common acceptable method for HAp synthesis is sintering, which was shown to produce HAp able to form a very tight bond with bone tissue [10]. After synthesis, HAp characterization is necessary in order to investigate the structure and properties of the material regarding its ability to meet set standards for different applications [10]. HAp characterization techniques are numerous and include XRD (X-ray diffractometry), XRF (X-ray fluorescence), FE-SEM (field emission scanning electron microscopy), and TEM (transmission electron microscopy) [11]. 

While HAp (Ca_10_(PO_4_)_6_(OH)_2_) is relatively simple to produce and possesses low toxicity, high biocompatibility and bioactivity, its intrinsic mechanical weakness (brittleness and powdery characteristics) often requires combination with other materials. These materials include natural polymers (chitosan, silk fibroin, collagen, hyaluronic acid), synthetic polymers (polylactic acid, polycaprolactone, poly-lactic-co-glycolide acid), [12] and different ions (magnesium, zinc, lithium and others) [6]. Also, it has been recognized that the HAp particle size is an important factor in the regeneration process, and that nano-hydroxyapatite (nHAp) with smaller (1–100 nm) particles possesses the most desirable traits, partly due to their huge surface-to-volume ratio and faster resorption followed by new bone formation [13]. In addition, similarity to numerous body proteins and ligands makes it easier for nHAp to interact with multiple receptors and cross cell membranes. However, a particle size under 10 nm is potentially lethal and reactive because of its high surface density and increased surface reactive electrons [14]. In order to ensure improved and accelerated bone regeneration, nHAp scaffolds are often enriched with different bioactive components, including stem cells, hormones, growth factors, exosomes, microRNAs and peptides [15]. 

Before translation to clinical contexts, potential bone scaffolds need to be assessed through reliable and comparable methods, including both in vitro and in vivo evaluation. While in vitro assays usually serve as a screen for host response prediction, in vivo animal experiments provide a wide range of parameters regarding the interaction of the scaffold with the host’s immune system and surrounding tissues as well, which acts as a mechanical environment affecting the implanted material [16]. However, due to variations concerning the type of animals used in different studies, sample sizes, defect sizes and locations, duration and method of regeneration assessment, etc., it is difficult to draw clear conclusions and ascertain the superior bone scaffolds. 

Previous systematic reviews regarding bone regeneration in in vivo studies have examined the use of scaffolds consisting of HAp alone [17], analyzed the effects of different bioceramic scaffolds (including HAp) in critical-sized defects [18], showed the superior characteristics of various scaffolds when combined with stem cells [19,20], and compared the application of nHAp alone or as a delivery system for drugs and bioactive molecules [21]. However, to the best of our knowledge, the application of scaffolds based on nHAp compared to nHAp scaffolds enriched with bioactive components for the regeneration of critical-sized bone defects in animal models has not been systematically reviewed to date.

In this review, we critically examined whether the enrichment of nHAp/polymer scaffolds with growth factors, hormones, polypeptides, microRNAs and exosomes improved new bone formation in vivo.

## 2. Materials and Methods

### 2.1. Study Protocol and Registration

This systematic review adhered to the guidelines set out in the Preferred Reporting Items for Systematic Reviews and Meta-Analyses (PRISMA) statement [22]. Additionally, the search methodology aligns with the PRISMA-S guidelines from 2021 [23]. The protocol for this review was registered with the International Prospective Register of Systematic Reviews (PROSPERO) with registration number CRD42024531511.

### 2.2. Focal Question

The specific PICO (Population, Intervention, Comparison, Outcome) question investigated was as follows: in experimental animal models with bone defects, does the utilization of a nano-hydroxyapatite-based scaffold enriched with bioactive molecules (specifically, growth factors, hormones, polypeptides, microRNAs, exosomes) enhance new bone formation compared to employing a scaffold solely based on nano-hydroxyapatite?

Population: experimental animal models with bone defects.
Intervention: utilization of a nano-hydroxyapatite-based scaffold enriched with bioactive molecules (growth factors, hormones, polypeptides, microRNAs, exosomes).
Comparison: employing a scaffold solely based on nano-hydroxyapatite.
Outcome: enhancement of new bone formation.

### 2.3. Eligibility Criteria

#### 2.3.1. Inclusion Criteria

- Randomized or non-randomized controlled experimental studies in animals with a minimum of two study groups and at least four animals/treatments per group.
- Control group of composite scaffolds that contain 30% *w*/*v* of nano-hydroxyapatite or more.
- At least one experimental group that used composite scaffolds containing 30% *w*/*v* of nano-hydroxyapatite or more, enriched with a bioactive molecule or molecules.
- Animal experiments that induced critical-sized bone defects to investigate bone regeneration.
- Studies on healthy, non-medically compromised animals.

#### 2.3.2. Exclusion Criteria

- In vitro studies, clinical studies, literature reviews, meta-analyses, and book chapters.
- Animal studies exclusively reporting ectopic models (such as subcutaneous or intramuscular).
- Absence of adequate control group.
- Treatment of periodontal defects, tooth extraction sockets.
- Research involving scaffolds loaded with chemotherapeutic agents, anti-inflammatory drugs, antibiotics, or ions.
- Research involving scaffolds loaded with stem cells.
- Studies written in Sinitic languages.

### 2.4. Information Sources and Search Strategy

Extensive literature searches were conducted from 28 March to 9 April 2023 across multiple electronic databases: Clarivate Analytics’ Web of Science (encompassing Web of Science Core Collection—WoS, Korean Citation Index—KCI, SciELO Citation Index—SCIELO, ProQuest™ Dissertations & Theses Citation Index, Grants Index), Scopus, and PubMed (including MEDLINE). The initial searches aimed to identify key terms, synonyms, and relevant controlled vocabulary (Medical Subject Headings—MeSH, available at https://www.ncbi.nlm.nih.gov/mesh/) and to evaluate various search strategies. A comprehensive search strategy (provided in Supplementary Table S1), developed collaboratively by an experienced medical librarian (J.J.) and the review team, underwent peer review following the PRESS guidelines [24]. Feedback from a second information specialist was incorporated before the final database search. Additionally, efforts were made to identify relevant unpublished materials such as research reports, conference papers, doctoral dissertations, and other grey literature through sources like OpenGrey (http://www.opengrey.eu), Google Scholar (first 100 results), and various digital repositories (e.g., Networked Digital Library of Theses and Dissertations—http://www.ndltd.org, Open Access Theses and Dissertations—https://oatd.org, DART-Europe E-theses Portal—DEEP—https://www.dart-europe.org/basic-search.php, and EThOS—Opening access to UK theses—https://ethos.bl.uk). Furthermore, to ensure comprehensiveness, backward and forward snowballing techniques were employed using citation indexes (WoS and Scopus) and Google Scholar. Searches were repeated during the final stages of manuscript preparation until 12 May 2024, confirming no new relevant trials were published subsequent to the initial literature review.

### 2.5. Study Selection and Data Extraction

The Rayyan QCRI platform [25] facilitated the importation of all literature search results to eliminate duplicates and begin screening. Initially, two independent investigators (D.M. and J.C.) thoroughly screened titles and abstracts to identify studies that met predetermined inclusion criteria. Papers that did not meet these criteria were excluded, and full texts of initially selected studies were retrieved for comprehensive evaluation. Authors of inaccessible papers were contacted via email to request the full texts. In the subsequent stage, the same two investigators independently assessed the full texts to determine relevance. Any discrepancies were resolved through consensus or consultation with a third investigator (M.J.K.).

Two independent reviewers (D.M. and J.C.) conducted the data extraction, with any discrepancies resolved by a third reviewer (M.L.), using a customized extraction form in MS Excel. Extracted details include the first author’s name and country, publication year, journal name, study design type, type and number of animals, characteristics of bone defect, type of composite scaffold, control groups, experimental groups, bioactive molecules, and observation periods.

### 2.6. Outcome Measures

#### 2.6.1. Primary Outcomes

Bone healing, reported as new bone formation measured by histomorphometry or micro-CT (µCT), and reported as bone mineral density, bone volume, and/or bone formation rate, served as the primary outcome.

#### 2.6.2. Secondary Outcomes

Secondary outcomes include the following: (1) bone healing described by qualitative measurements of histological and radiological assessments; (2) monitoring of complications and adverse events associated with the biomaterials employed; (3) investigation of scaffold components and the method of loading bioactive molecules to the scaffold.

### 2.7. Quality Assessment and Risk of Bias Analysis

Two reviewers (D.M. and J.C.) independently evaluated the quality of the studies according to the ARRIVE (Animals in Research: Reporting In Vivo Experiments) guidelines [26]. The criteria assessed included an ethical statement, experimental procedures, use of experimental animals, randomization, allocation concealment, sample size calculation, completeness of information, blinding of the evaluator, and financial conflicts of interest.

The studies were assessed for bias using the SYRCLE method [27], which involves evaluating ten items with judgments of yes/no/unclear to determine the risk of bias. Studies were categorized as having a high risk of bias if they received a rating of “no” for at least two items. On the other hand, studies were considered to have a minimal chance of bias if at least seven criteria were evaluated as “yes” and none were evaluated as “no”. Otherwise, the studies were classified as having a moderate risk of bias.

### 2.8. Data Synthesis and Statistical Analysis

Because of the heterogeneity in study protocols, biomaterials utilized, methods of outcome assessment, outcome measures, and duration of follow-up, a meta-analysis could not be conducted. Therefore, qualitative data extracted from each study were compiled and synthesized into analytical tables.

## 3. Results

### 3.1. Study Selection

**Figure 1:**
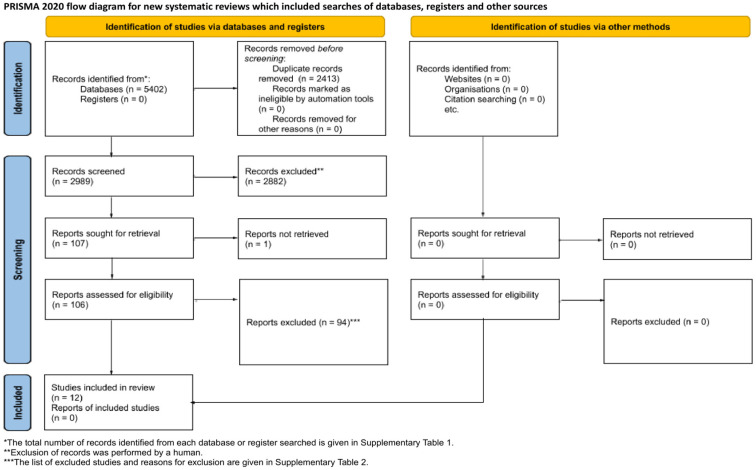
PRISMA flow diagram. From: Page M.J., McKenzie J.E., Bossuyt P.M., Boutron I., Hoffmann T.C., Mulrow CD, et al. The PRISMA 2020 statement: an updated guideline for reporting systematic reviews. BMJ 2021;372:n71 [22].

The PRISMA flowchart in Figure 1 details the applied literature search strategy. Initially, 5042 studies were identified through the primary search, of which 2413 were removed as duplicates. The subsequent screening of the titles and abstracts led to the exclusion of 2882 additional studies. A total of 106 studies were then subjected to full-text evaluation. After a thorough examination, 94 studies were excluded based on the reasons outlined in Supplementary Table S2. Ultimately, 12 studies [28,29,30,31,32,33,34,35,36,37,38,39] met the criteria and were included in the present systematic review.

### 3.2. Study Characteristics

The eligible articles included in this review were original papers published between 2009 and 2023. Among them, four studies were performed using rabbits [33,34,37,39], while eight studies used rats as animal models [28,29,30,31,32,35,36,38]. Animals had bone defects created either on the calvaria [28,29,30,31,32,35,36], femur [38], mandible [34,37], or radial bone [33,39] (Table 1). Most of the studies used calvaria critical-sized defects with a diameter ranging between 5 and 7.5 mm in rats, and radial bone defects in rabbits, with a diameter of 7 mm, created by drilling circular, unilateral, or bilateral defects. A femoral super critical-sized defect, 10 × 5 × 3 mm, in rats, was used in the study of Zhao et al. [38], while Zhang et al. [34] investigated bone healing after creating super-critical bilateral mandibular defects of the dimensions 10 × 5 × 5 mm.

Histological evaluation was the most commonly used method (n = 10) to assess bone healing, followed by micro-computed tomography analysis (n = 7), radiographic evaluation (n = 5), and histomorphometric analysis (n = 4). Other less commonly used methods included immunohistochemistry and three-dimensional computer tomography (3D CT). The follow-up periods ranged from 1 to 12 weeks. Most of the studies had two observation periods, with 4 and 8 weeks being the most frequently used. Two out of twelve studies reported a single observation period, while two studies had three observation periods.

As composite scaffolds with nHAp have numerous possible combinations of constituents, the scaffolds’ composition and nHAp proportion were analyzed and summarized in Table 2. A variety of constituents were used in the studies, while one half of the studies used a combination of collagen and nHAp. Other studies used chitosan, hyaluronic acid, glycerol, poly-L-lactic acid, gelatine, polyamide 66, or silk fibroin as an addition to nHAp. The proportion of nHAp in the composite scaffolds used in the reviewed articles was between 30% and 82.5%.

Various bioactive components were used to accelerate and further stimulate bone regeneration. Half of the studies used BMP2, human recombinant BMP2 (rhBMP2) [33], and/or its synthetic variants, P24 [28,32], P28 [30], and P17-BMP2 [34]. Among them, one study used a combination of BMP2 and VEGF [29]. Three other studies used microRNA [35] and/or microRNA inhibitors [31,36]. The remaining studies used insulin [37], RGD peptide [38], and BMSC exosomes [39] as bioactive molecules to investigate accelerated bone regeneration.

Different methods were chosen to deliver the bioactive components to the scaffolds. Most often, the scaffold was immersed into a bioactive component solution. Several studies used micro- and nano-sized carriers loaded with bioactive components, such as PLGA microspheres [28] and nanospheres [37], and nHAp particles [31,36]. One study used the preparation of scaffolds by applying a layer of fibrin glue on the surface [33], while another study prepared the scaffold surface with polydopamine coating [39], before immersion in a bioactive component solution. 

Prior to in vivo implantation, most of the studies investigated the in vitro release of bioactive components from the scaffold into the surrounding area. When microspheres [28], fibrin glue [33], and polydopamine coating [39] were used as delivery systems, continuous release was observed for a prolonged period. On the other hand, when bioactive components were soak-loaded into the scaffold, predominantly rapid release in the first few days was observed, followed by low release over prolonged periods [30,32,35,37]. In the research of Castano et al. [31], no in vitro release was investigated, but a one-week in vivo pilot study was conducted to observe the delivery of antagomiR-133a to the bone defect site, and its possible dispersion to the surrounding area. The study demonstrated that the antagomiR-133a, which was labeled with fluorescent markers, was evenly distributed and remained within the scaffolds. This was followed by a significant infiltration of host cells. This suggests that the scaffold-based delivery system prevented the breakdown of antagomiR-133a and prolonged its lifespan before being removed from the implant site.

Even though the initial search included all in vivo studies, only two animal models met the criteria for the final analysis. Thus, the results are presented corresponding to included t animal models.

#### 3.2.1. Characteristics of the Studies with Rabbit Animal Models

Nanostructured HAp was combined with gelatine [33], collagen [34,37], and silk fibroin and chitosan [39] so tridimensional composite scaffolds could be obtained. The scaffolds based on nHAp generally stimulated bone regeneration, and none of the studies reported adverse reactions during the healing periods. The bioactive components used to further stimulate and accelerate the formation of the new bone generally had significant stimulative effects. The observation periods varied from 2 to 12 weeks. The main findings of the studies in rabbits are summarized in Table 3.

In the study of Zhang et al. [34], the regenerative effects of BMP2 addition, in low and high concentrations, were investigated on the mandibular critical-sized defect. The overall results signified more evident new bone formation when BMP2 was added to the nHAp scaffolds. The active early formation of new bone was reported as early as after two weeks in both BMP2 groups. After four weeks a higher concentration of BMP2 (10 mg/g) stimulated bone union, complete spongiosa formation, and early detection of the cortex. BMP2 was used in another study [33] as recombinant human BMP2 for the enhancement of segmental radial defect healing. Twelve weeks after surgery, complete bone defect reparation occurred in the rhBMP2 group, while partial regeneration occurred in the rhBMP2-free group. Another study [39] chose a 12-week observation period for the healing of the radial bone defect. In addition to the nHAp scaffold, they employed exosomes isolated from bone marrow mesenchymal stem cells (BMSCs). In the exosome-free group, the bone defect area was partially repaired, while the rate of new bone formation was significantly higher (*p* < 0.01), and the new bone resembled the normal, surrounding bone tissue in scaffold doped with exosomes. When insulin was used as a bioactive component [37], in the mandibular defect bone model, scaffold pore channels were filled with bone matrix 4 weeks post-implantation in the insulin-enriched group, while in the insulin-free group, no bone formation was observed. Eight weeks after implantation, mature lamellar bone was observed in the insulin-enriched group, while thin trabecular bone formations were reported in the controls.

#### 3.2.2. Characteristics of the Studies with Rat Animal Models

In the studies with rats as animal models, nHAp was combined with hydroxypropyl chitosan [28], hyaluronic acid and demineralized bone matrix [29], glycerol [30], collagen [31,32,35,36], and polyamide 66 [38], so tridimensional composite scaffolds could be obtained. The scaffolds based on nHAp generally stimulated bone regeneration, and none of the studies reported an adverse reaction during the healing periods. The bioactive components used to further stimulate and accelerate the formation of the new bone generally had a significant stimulative effect. The observation periods varied from one to 12 weeks. The main findings of the studies on the rat animal models analyzed in this review are presented in Table 4.

In the study of Cai et al. [28], a synthetic form of BMP2, P24, was used as an addition to the scaffolds to investigate its potential to accelerate the healing of the bone defect. On week four post-implantation, osteocalcin was expressed only in the group with P24 addition. Another study [30] investigated the addition of BMP2 and P28 to scaffolds, and their effect on bone regeneration. The overall results signified more evident new bone formation in the scaffolds loaded with BMP2 or P28 in comparison to the control nHAp scaffold. Interestingly, no significant differences were observed between the scaffolds loaded with BMP2 and those loaded with its synthetic variant, P28. The other study [32] compared the stimulative effect of P24 and rhBMP2 on bone regeneration. The six weeks’ post-surgery volume of the repaired defect reached 10.2% in the control, and 42% in the P24 and 51.8% in the rhBMP2 group. Twelve weeks after surgery, the repaired areas closely resembled the surrounding host bone in the experimental groups. In the observed periods, no significant difference was observed between the P24 and rhBMP2 groups. 

Townsend et al. [29] investigated the regenerative potential of nHAp, demineralized bone matrix (DBM), and decellularized cartilage (DCC), combined with hyaluronic acid (HA) in the form of colloidal gels. As an addition, BMP2 and VEGF growth factors were included. The main results of the µCT analysis revealed significant regenerated bone volume in the HA/nHAp-BMP2 and HA/nHAp/DCC groups compared to an empty defect. The addition of VEGF and the combination of DBM and DCC with growth factors did not stimulate significant generation of new bone. 

Castano et al. [31] reported the localized release of antagomiR-133a loaded onto the nHAp scaffold to the implant site at 1 week after surgery, with evident calcium deposits in the antagomiR-133a group. Furthermore, a 4-week post-surgery 2.2-fold bone volume increase was revealed in the antagomiR-133a group in comparison to the control scaffold. Similar to these results, when the nHAp-miR-26a nanoparticles were loaded on an nHAp scaffold [35], a 1.7-fold increase of new bone formation was reported 4 weeks post-implantation, and a 1.6-fold increase over the microRNA-free scaffold at the end of week 8. While the previously mentioned studies used a microRNA inhibitor or microRNA alone, a study from 2023 [36] employed the use of both, a miR-210 mimic and miR-16 inhibitor. At four weeks’ post-implantation, the dual-microRNA-loaded scaffold induced more than double the bone volume and increased vessel recruitment by 2.3-fold over the microRNA-free scaffold.

Zhao et al. [38] loaded RGD peptide onto the nHAp scaffold, which stimulated a significantly greater volume of regenerating bone over the control scaffold. The authors did not specify the main differences after 8 and 12 weeks in particular, but the overall significance of the RGD addition to the scaffold for the whole observation period was noted.

## 4. Discussion

Nanostructured HAp composite scaffolds modified by various bioactive components could be a promising strategy for bone repair and regeneration by accelerating the healing of bone defects [40]. Several previous narrative reviews have discussed the use of bioactive factors to enhance bone regeneration [15,40,41,42]. As various scaffolds and bioactive factors have been employed in in vitro and in vivo studies, this systematic review was conducted to gain a further insight into the matter. 

The aim of this systematic review was to critically assess whether the enrichment of nHAp-based scaffolds with bioactive components improves new bone formation in animal models. The overall results of all 12 included studies suggested that the addition of bioactive components accelerates bone regeneration of critical-sized defects compared to nHAp-based scaffolds alone. However, while the results of the different studies were in accordance, the lack of standardization of various variables (types of animals used, sample sizes, location and size of defects, percentage of nHAp, polymers and bioactive components in scaffolds, methods and duration of bone regeneration assessment) did not allow us to perform a meta-analysis.

The most frequent reasons for study exclusion were as follows: a small percentage of nHAp (less than 30%), the absence of an adequate control group or solely bioactive molecule group as an addition to the basic scaffold, and the use of cells as enrichment onto scaffolds. While the use of cells loaded onto scaffolds is widely explored in in vivo studies for bone regeneration evaluation, these studies were excluded from this review due to the existence of previous systematic reviews that have already dealt with this issue [19,20]. 

It should also be mentioned that in vivo studies involving larger animals (sheep, dogs, non-human primates, etc.), provide better insight into scaffold efficacy since they are a closer match to human bone [18], but due to ethical concerns and cost, they are conducted less frequently. Thus, only studies on rabbit and rat models were able to satisfy the eligibility criteria and be included in our systematic review.

Bone morphogenetic protein 2 (BMP2), a member of the transforming growth factor-β family, has a valuable role in tissue growth and regeneration [43,44,45]. Our analysis showed that among the various bioactive components used as scaffold adjuvants, BMP2, as well as recombinant human BMP2 (rhBMP2), and the related synthetic peptides P17-BMP2, P24, and P28 were most frequently present. The repeated use of BMP2 and its synthetic forms in research on bone regeneration is expected, since members of the bone morphogenic protein (BMP) family are known to be essential for osteogenesis, with numerous clinical and preclinical studies demonstrating their osteoinductive capacity [46,47]. The results of the studies included in our review furthermore validated the significance of its use in bone regeneration. BMP2 was shown to stimulate peripheral bone growth [29], and significantly influence higher bone mineral density (BMD) [30] in rat calvaria defects. When VEGF was used independently or in combination with BMP2 [29], it did not significantly affect bone regeneration; however, it did stimulate neoangiogenesis.

Still, the use of natural BMP2 has several disadvantages, such as a high cost, unreliable activity in living systems, and possible unfavorable reactions when loaded into scaffolds [48]. In addition, the use of rhBMP2 raises safety concerns related to potential toxicity and immunogenicity [49]. Also, the efficacy of rhBMP2 in bone regeneration is predominantly dependent of the material chosen to be a delivery system to the local defect site [50]. In our analysis, rhBMP2 was associated with a higher percentage of repaired cranial defects in rats [32] and the superior regeneration of radial rabbit defects [33]. 

Many BMP2 synthetic peptides were designed to be used as a substitution for natural BMP2 in bone defect studies [51,52]. As a synthetic form, P17-BMP2 stimulated rapid bone generation in rabbit mandibular defects [34]. Interestingly, when a higher dose of P17-BMP2 was used, it resulted in higher radiographic and histological scores compared to a lower dose. P24 contributed to more evident bone regeneration [28,32] and P28 to higher BMD [30] in rat cranial defects. All of the included studies showed no advanced effect of natural BMP2 over its synthetic versions, suggesting their possible use in further clinical investigations.

Another bioactive component for scaffold doping, which has been receiving great attention in recent years, are microRNAs. These small non-coding RNAs controlling gene expression have various functions, and their role in osteogenesis has been well documented [53]. Due to their ability to influence both positive and negative regulators of osteogenesis, microRNAs’ effects may contribute to the promotion as well as inhibition of bone formation [53]. This justifies scaffold doping with not only microRNA mimics but also antagonists (antagomiRs), depending on their stimulative or inhibitory osteogenesis effects. All three studies with microRNAs included in this review, using either mimics, antagomiRs, or both, have shown their positive effects on bone regeneration. Still, some microRNAs proved to be more potent than others. Sadowska et al. demonstrated that miR-26a-enriched scaffolds had numerous advantages in osteogenesis stimulation compared to miR-26a-free scaffolds [35], while Castano et al. have shown similar effects on bone formation by using antagomiR 133a [31]. A combination of both microRNA mimics (miR-210) and antagonists (antagomiR-16) for scaffold doping stimulated bone repair augmentation, although without statistical significance [36].

Exosomes, small (30–150 nm) extracellular vesicles secreted from various cells, are shown to be essential in intercellular communication [54]. Although exosomes have poor bioavailability in their free form, loading them onto scaffolds overcomes this shortcoming [54]. Also, due to their nanoparticle nature, they can be incorporated into scaffolds more easily than cells [55]. In this way, all the potential of their parent cells is retained since they contain both RNA and proteins. On the other hand, self-replication or auto-immune reactions, often drawbacks of cell use, are avoided [55]. While only one study with the use of exosomes was included in the final analysis, based on the reported results, it could be stated that exosome-loaded scaffolds are a promising avenue in bone regeneration [39]. Further studies should provide insight into the promising clinical use of exosome in bone regeneration.

Insulin, a hormone that regulates body energy balance, is also involved in bone metabolism. In recent years, it has been accepted as an osteogenesis induction factor. BMSCs responded to exogenous insulin addition with increased proliferation due to functional receptors for insulin found on their membrane [56], and the stimulation of alkaline phosphatase [57], associated with PI3-K/Akt signaling pathway upregulation [58]. In in vivo studies, it was reported that insulin promoted bone regeneration in calvaria defects by the promotion of osteoblast proliferation [59], and the regulation of osteoclast activity [60]. However, the short life of insulin in the body and the absence of a defined adequate dosage still limits its application in bone regeneration. As the half-life of free insulin is about 20–30 min [61], the use of insulin in in vivo studies depends on a methodology that can enable controlled release with prolonged activity. PLGA nanospheres used to deliver insulin to bone defect sites provided five days of sustained release of insulin [37]. Significant bone regeneration was reported in the insulin group, indicating a possible sustainable system for in vivo insulin delivery.

RGD peptides, a functional group present in the extracellular matrix, are designed for further peptide materials modification. They are reported as essential regulators of cell growth, involved in multiple biological processes. After binding to integrin receptors, they affect cells’ migration, proliferation, and differentiation [62,63]. In the study of Huang et al. [64], the RGD peptides were introduced into the peptide sequence of RADA16, and D-RADA16-RGD nano-fibers were further incorporated into the nHAp scaffold. RGD peptides caused new bone formation that completely resembled the surrounding healthy tissue, indicating the need for further research on RGD peptides’ potential to stimulate bone regeneration.

Another factor that could affect accelerated bone regeneration is the slow and sustained release of bioactive molecules. The surface modification of composite scaffolds, or the loading of bioactive molecules into nano- and microspheres adsorbed to scaffolds, could provide adequate and prolonged delivery in a defect site [40]. Most of the analyzed studies added bioactive components through soak-loading the scaffolds into bioactive component solution. As a result, several studies reported the very rapid release on the first day, while the later release remained low [30,32,35,37]. Huge amounts of bioactive components released quickly could have an adverse effect on tissue healing and bone regeneration, cause heterotopic ossification, and inhibit bone formation, depending also on the concentration of bioactive components [47]. Thus, the slow and continuous release of growth factors at the site of implantation could be of the utmost importance in the bone healing process [65,66]. 

Micro-/nanospheres can be used as carriers incorporated into the scaffold, so bioactive components could be locally delivered to the bone defect site. Growth factors could be encapsulated in polymer microspheres as an intermediate delivery tool that serves as a protective barrier during scaffold production so the biological activity remains intact. When implanted, bioactive molecules are stably and sustainably released slowly at the target site, so effective bone tissue repair could be promoted over a prolonged period [67,68,69,70]. When P24 was delivered loaded onto PLGA microspheres [28], it significantly promoted bone regeneration in the 4- and 8-week period post-implantation. Lui et al. [33] used fibrin glue mixed with rhBMP2 and infused it into the scaffold. Slow and sustained release was achieved for 40 days, and the segmental radial bone defect (15 mm) was repaired completely 12 weeks after implantation in the rhBMP2 group. This implies that even oversized bone defects could be successfully regenerated with bioactive component-enriched nHAp scaffolds that stimulate and accelerate bone regeneration over a prolonged period. 

Natural mucin, polydopamine (PDA), has the ability to form covalent and noncovalent bonds on material surfaces which enables its stable adherence to the surface of numerous inorganic and organic materials [71,72]. Also, PDA in a weakly alkaline environment can oxidize and form a polymer, that further efficiently adsorbs bioactive components. In an alkaline environment of nHAp scaffold, this polymer could improve the functional characteristics of the scaffold [73,74]. When PDA was used for exosome loading onto an nHAp scaffold [39], the sustained release of exosomes was obtained, providing the complete restoration of over-critical bone defects. The newly formed bone resembled the surrounding healthy bone tissue, indicating the most promising use of PDA and exosomes in bone regeneration. 

Hydrogels have been widely used for growth factor delivery due to their injectable, easy chemical modification. Since hydrogel degrades in time, active components are continuously being released into the surrounding environment. Due to hydrogel permeability, nearby tissues and cells can absorb components from hydrogel [75,76,77]. Hydrogel and colloidal gel were used in two of the analyzed studies [28,29], where significant bone repair was linked with slow release of growth factors.

## 5. Conclusions

In conclusion, growth factors, hormones, polypeptides, microRNAs, and exosomes were reported to stimulate accelerated bone regeneration in a variety of bone defect models, showing better results than bone grafting with nHAp scaffolds alone. The results indicated that composite materials based on nHAp are excellent candidates as bone substitutes, while nHAp scaffold enrichment further accelerates bone regeneration. Regarding the use of growth factors, promising evidence suggests that the addition of BMP2, rhBMP2, and related synthetic peptides (P17, P24, and P28) further enhances new bone formation in in vivo studies, while the use of VEGF did not promote further mineralized tissue production. Still, it contributed to neovascularization.

Nevertheless, because of the significant differences in the composition of the scaffolds, the size and characteristics of the bone defects, the concentration and type of bioactive substances, the delivery methods, and the duration of the follow-up, it is not possible to draw definitive conclusions about the clinical efficacy of nHAp composite scaffolds for enhancing bone regeneration.

Therefore, the standardization of animal models should be performed in order to clearly define the most significant parameters of in vivo studies (size sample requirements, critical-sized defects depending on animal type, observation periods, and assessment methods followed by defined quantitative measurements of new bone formation) to future researchers. Only in this way could the adequate comparison of findings from different in vivo studies be possible, further advancing our knowledge on bone regeneration and enabling its translation to clinical settings. The results of this study have opened a promising avenue for further clinical research into bioactive components’ addition to the well-established nHAp scaffolds and their impact on accelerated bone regeneration.

## Figures and Tables

**Table 1:** Distribution of defect types among the included studies.

Animal	Study Model	Number of Studies	References
Rabbit	Segmental radial defect	2	[33,39]
	Mandibular defect	2	[34,37]
Rat	Calvaria defect	7	[28,29,30,31,32,35,36]
	Cylindrical femoral defect	1	[38]

**Table 2:** Bone scaffold composition and main properties of bioactive molecules.

Reference	Composite Scaffold	nHAp Content (%)	Form	Bioactive Component Concentration	Method of Binding to Scaffold	Release of Bioactive Molecules In Vitro
[28]	nHAp/HPCS/PLGA-P24 microspheres	33	Hydrogel	P24 (not specified conc.)	P24-loaded PLGA microspheres	Continuous release for 60 days
[29]	HA/nHAp/ECM (DCC or DBM)	82.5	Colloidal gel	25 μg/mL BMP2, VEGF	Mixed in PBS with material powder	/
[30]	nHAp/glycerol	30	Disk	3 mg of P28, or 3 μg of BMP2	Peptide suspension dropped on the scaffold	Rapid release, particularly in first 12 h; latter release rate remained low
[31]	collagen/nHAp	50	Disk	50 μL antagomiR-133a particles	Soak-loaded with antagomiR-133a or blank nHAp particles	/ *
[32]	nHAp/collagen/PLLA	~30%	Disk	3 mg P24, 1 μg rhBMP2	Materials were impregnated into the solution	Very rapid release rate on the first day, decreased with time
[33]	gelatine/nHAp/FG	80%	Disk	10 μg rhBMP2	FG mixed with rhBMP2 infused into scaffold	Overall release slow and sustained, ended on day 40
[34]	nHAp/collagen/PLLA	~30%	Disk	2 and 10 mg/g P17-BMP2	Scaffold impregnated into P17-BMP2 solution	/
[35]	collagen/nHAp	50%	Disk	1 μg of miR-26a nanoparticles	Soak-loaded into scaffold	Initial release with a 28-day plateau phase
[36]	collagen/nHAp	50%	Disk	10 nM miR-210 mimic and 10 nM antagomiR-16	Soak-loaded with miRNA-nHAp complexes or blank nHAp particles	/
[37]	collagen/nHAp	45 ± 5	Disk	0.5% insulin	Immersed into insulin-loaded PLGA nanosphere suspension	Initial high release (46% within 24 h), and constant slow release over next 5 days
[38]	nHAp/PA66	40	Disk	5 mg/mL D-RADA16-RGD peptide	Immersed in D-RADA16-RGD solution	/
[39]	SF/CS/nHAp/PDA	33	Disk	1.8 μg/μL exosomes from BMSCs	Polydopamine coating immersed in exosome solution	Sustained release until the 21st day

HPCS—hydroxypropyl chitosan, HA—hyaluronic acid, PLLA—poly (L-lactic acid), FG—fibrin glue, PA66—polyamide 66, SF—silk fibroin, CS—chitosan, PDA—polydopamine coating, PLGA—Poly(lactic-co-glycolic acid), BMP2—bone morphogenetic protein 2, rhBMP2—recombinant human BMP2, VEGF—vascular endothelial growth factor, BMSCs—bone marrow mesenchymal stem cells, * in vivo release of bioactive component.

**Table 3:** Summary of main methods and results of studies in rabbits.

Ref.	Sample Size (Animals Number)	Defect	Control Group(s)	Bioactive Components	Treatment Group(s)	Duration (Weeks)	Assessment Method(s)	Main Findings
[33]	n = 5 (45)	15 mm segmental radial defect	Empty, gelatin/nHAp/FG	rhBMP2	Gelatin/nHAp/FG-rhBMP2	4, 8, 12	X-ray, H&E	Week 4: control—callus started to form while implanted material degraded; rhBMP2 group—bone tissue confluent with implanted material, and a callus started to form. Week 8: control—degradation of implanted material, while margins and bone tissue become cloudy; rhMBP2 group—massive callus formed, boundary of implanted material and bone tissue become cloudy. Week 12: control—partial recanalization of medullary cavity and capillary regeneration; rhBMP2 group—complete regeneration with full recanalization of medullary cavity.
[34]	n = 5 (20)	10 × 5 × 5 mm, bilateral, mandibular defect	Empty, nHAp/collagen/PLLA	P17-BMP2	nHAp/collagen/PLLA/P17-BMP2 2 mg or 10 mg	2, 4	X-ray, H&E	Week 2: control group—formation of fibrous union and small blood vessels; for BMP2 (2 mg/g) and BMP2 (10 mg/g) groups, there was fibrous union and active early new bone formation. Week 4: control group—fibrous union and active new bone formation; for BMP2 (2 mg/g)—bone union and spongiosa formation; and for BMP2 (10 mg/g)—bone union, complete spongiosa formation and early detection of cortex detected.
[37]	n = 5 (40)	10 × 5 × 3 mm, critical-sized mandibular defect	Collagen/nHAp, collagen/nHAp/PLGA	Insulin	Collagen/nHAp/PLGA-insulin	4, 8	µCT, histomorphometry	Week 4: collagen/nHAp/PLGA group—no observed bone formation; collagen/nHAp/PLGA-insulin—bone matrix in scaffold pores. Week 8: collagen/nHAp/PLGA group—thin trabecular bone formations seen; collagen/nHAp/PLGA-insulin—mature lamellar bone with abundant mineralized area. The amounts of bone formed (BV/TV) in collagen/nHAp/PLGA and collagen/nHAp/PLGA-insulin groups were 30.3%, and 46.6%, respectively.
[39]	n = 6 (24)	15-mm long, 3-mm-diameter cylindrical radial defect	Empty, SF/CS/nHAp, SF/CS/nHAp/PDA	BMSC exosomes	SF/CS/PDA-Exosomes	12	µCT, histomorphometry, H&E	In SF/CS/PDA—partially repaired bone defect, lower bone mineral density and partial obstruction of bone marrow cavity observed, compared with normal bone tissue. In SF/CS/PDA-Exosomes—repaired bone defect area resembled normal bone tissue, bone marrow cavity completely recanalized with continuous bone cortex. The rate of new bone formation was significantly higher (*p* < 0.01), as well as Col-I and CD31 expression (*p* < 0.05).

X-ray—radiographic, µCT—micro-computed tomography, H&E—hematoxylin and eosin staining, Col-I—collagen I.

**Table 4:** Summary of main methods and results of studies in rats.

Ref.	Sample Size (Animals Number)	Defect	Control Group(s)	Bioactive Components	Treatment Group(s)	Duration (Weeks)	Assessment Method (s)	Main Findings
[28]	n = 10 (30)	5 mm diameter unilateral calvaria defect	nHAp/HPCS, nHAp/HPCS/PLGA	P-24	nHAp/HPCS/PLGA-P24	4, 8	IHC, µCT, H&E	Week 4: OCN only expressed in CD/n-HA/PLGA-P24. On µCT in CD/n-HA and CD/n-HA/PLGA, poorly stimulated bone regeneration, while in CD/n-HA/PLGA-P24, bone island observed. Week 8: bone regeneration was slow in CD/n-HA and CD/n-HA/PLGA, but in CD/n-HA/PLGA-P24, recovery was evident.
[29]	n = 5 (36)	7.5 mm diameter unilateral calvaria defect	Empty, HA/nHAp, HA/nHAp/DBM, HA/nHAp/DCC	BMP2, VEGF	HA/nHAp-BMP2 or VEGF, HA/nHAp-BMP2-VEGF, HA/nHAp/DBM-BMP2 or VEGF, HA/nHAp/DCC-BMP2 or VEGF	8	µCT, H&E	Active bone formation with evident encapsulation of conglomerated particles was observed in all groups, with no differences between groups. In HA/HAp/DCC group and the BMP2 groups, new bone formation in periphery with ingrowth into defect site was significant compared to VEGF groups. Addition of VEGF contributed to the formation of thicker soft tissue bridging the defect site compared to other groups.
[30]	n = 4 (32)	5 mm × 2 mm full thickness calvaria defect	Empty, nHAp/glycerol	P28, BMP2	nHAp/glycerol-P28, nHAp/glycerol-BMP2	6, 12	µCT, H&E, histomorphometry	For each time point, newly formed bone was more evident in groups with BMP2 or P28 than in nHA/glycerol. Significantly higher BMD was observed in groups with BMP2 or P28 than in nHA/glycerol at week 6 and 12. No significant differences observed in BMD between scaffolds loaded with BMP-2 and those with P28.
[31]	n = 8 (24)	7 mm circular trans osseous calvaria defect	Empty, collagen/nHAp/empty nHAp particles	antagomiR-133a particles	Collagen/nHAp-Dy547-tagged antagomiR-133a particles	1, 4	µCT, H&E, histomorphometry	Week 1: increased deposition of large calcium deposits in antagomiR-133a-scaffolds. Week 4: antagomiR-133a-scaffold yielded 2.2-fold increase over antagomiR-free scaffolds (*p* = 0.17). Residual scaffold identified in both groups. New bone observed in antagomiR-133a scaffold, while in antagomiR-free tissue appeared predominantly granular. Histomorphometry—antagomiR-133a-scaffold 70% increase versus antagomiR-free (*p* = 0.0108).
[32]	n = 5 (30)	5 mm in diameter, unilateral, full thickness cranial defect	nHAp/collagen/PLLA	p24, rh-BMP2	nHAp/collagen/PLLA-P24, nHAp/collagen/PLLA-rhBMP2	6, 12	X-ray, 3D CT, H&E	Week 6: the gray values of P24 and rhBMP2 groups significantly higher than those of control, but P24 and rhBMP2 groups differed only slightly. Control had only 10.20% defect volume repaired, while in P24 and rhBMP2 groups, it reached 42.00% and 51.80%, respectively. Week 12: the respective repaired percentages of control, P24, and rhBMP2 were 32.40%, 84.40%, and 86.60%.
[35]	n = 8 (16)	7 mm diameter, unilateral, trans osseous calvaria defect	Collagen/nHAp	miR-26a	Collagen/nHAp-miR-26a nanoparticles	4, 8	X-ray, H&E	Weeks 4 and 8: miR-26a-scaffold induced formation of new bone by 1.7-fold and 1.6-fold increase over microRNA-free scaffold; also, there was a greater increase in bone volume compared to the microRNA-free scaffold, by 1.8-fold and 1.9-fold, respectively. Week 8: Although the results are not significant, miR-26a-activated scaffolds enhanced bone bridging, reduced the defect by 0.3-fold compared to microRNA-free scaffold. Enhanced vasculogenesis in miR-26a-scaffold compared to microRNA-free group (*p* = 0.095).
[36]	n = 8 (24)	7 mm diameter, unilateral, trans osseous calvaria defect	Empty, collagen/nHAp/empty nHAp particles	miR-210/16	Collagen/nHAp-miR-210/16 dual complexes	4	X-ray, H&E	MiRNA-free group—residual scaffold evident with appearance of predominantly granular tissue, with some bone infilling, while in miR-210/16-group, organized de novo bone seen clearly, with calcified tissue, signs of bridging and enhanced thickness. MiR-210/16 group achieved over twice as great bone volume as miRNA-free group (*p* = 0.029). Enhanced neovascularization in miR-210/16 group with significantly higher number of vessels and 2.3-fold increase in comparison to miRNA-free scaffolds
[38]	n = 6 (24)	10 × 5 × 3 mm, critical-sized mandibular defect	nHAp/PA66	D-RADA16-RGD peptide	nHA/PA66/D-RADA16-RGD	8, 12	µCT	Weeks 8 and 12: D-RADA16-RGD group demonstrated a significantly greater volume of regenerated bone, and a ratio of BV/TV in the new formed bone (*p* < 0.05). However, there were no significant differences in trabecular number at the 12-week timepoint (*p* = 0.114), and one reason for this may be the value of them reached values of healthy bone for the D-RADA16-RGD group.

DBM—demineralized bone matrix, DCC—decellularized cartilage, BMD—bone mineral density, IHC—immunohistochemistry.

## Data Availability

The data presented in this study are available on request from the corresponding authors.

## Supplementary Materials

The following supporting information can be downloaded at https://www.mdpi.com/article/10.3390/biomimetics9070386/s1, Table S1: Electronic databases and search strategy; Table S2: Excluded studies and reasons for exclusion.
