# Supplementary material for: The Impact of Nano-Hydroxyapatite Scaffold Enrichment on Bone Regeneration In Vivo—A Systematic Review
Source: Biomimetics (Basel). 2024 Jun 25;9(7):386. doi: 10.3390/biomimetics9070386 (PMC11274561; doi:10.3390/biomimetics9070386)
Supplement: Supplementary file 1 [file biomimetics-09-00386-s001.zip › Supplementary Table S1.pdf]

Supplementary Table S1. Electronic Databases and Search Strategy [April 9, 2024]

Database (n)		Search strategy #1 AND #2
WoS (n=1,713)<br>ProQuest™ (n=70)<br>Grants Index (n=19)<br>KCI (n=18)<br>SciELO (n=5)<br>Total (n=1,809)<br>https://www.webofscience.com	#1	TS=(((Bone OR osteochondral) NEAR/2 (Repair OR regenerat* OR tissue OR defect$ OR engineer*)) OR osseointegration OR osteoinduction OR osteoregenerat*) (n=346,378)
	#2	TS=(((nano-hydroxyapatite OR nanohydroxyapatite OR nanoHAP OR nano-HA OR nano-HAP OR n-Hap OR nHA OR n-HA OR nHAP OR “HAp NPs”) NEAR/2 (scaffold$ OR composit*)) OR ((nanomolecule$ OR nanoparticle$) NEAR/1 (hydroxyapatite))) (n=4,817)
Scopus<br>(n=1,681)<br>https://www.scopus.com	#1	TITLE-ABS-KEY (((Bone OR osteochondral) W/2 (Repair OR regenerat* OR tissue OR defect* OR engineer*)) OR osseointegration OR osteoinduction OR osteoregenerat*) (n=166,371)
	#2	TITLE-ABS-KEY (((nano-hydroxyapatite OR nanohydroxyapatite OR nanoHAP OR nano-HA OR nano-HAP OR n-Hap OR nHA OR n-HA OR nHAP OR “HAp NPs”) W/2 (scaffold* OR composit*)) OR ((nanomolecule* OR nanoparticle*) W/1 (hydroxyapatite))) (n=4,447)
PubMed<br>(n=1,912)<br>https://pubmed.ncbi.nlm.nih.gov/	#1	"Bone Regeneration"[Mesh] OR (((Bone[Title/Abstract] OR osteochondral[Title/Abstract]) AND (Repair[Title/Abstract] OR regenerat*[Title/Abstract] OR tissue[Title/Abstract] OR defect*[Title/Abstract] OR engineer*[Title/Abstract])) OR osseointegration[Title/Abstract] OR osteoinduction[Title/Abstract] OR osteoregenerat*[Title/Abstract]) (n=234,189)
	#2	((nano-hydroxyapatite[Title/Abstract] OR nanohydroxyapatite[Title/Abstract] OR nanoHAP[Title/Abstract] OR nano-HA[Title/Abstract] OR nano-HAP[Title/Abstract] OR n-Hap[Title/Abstract] OR nHA[Title/Abstract] OR n-HA[Title/Abstract] OR nHAP[Title/Abstract] OR "HAp NPs"[Title/Abstract]) AND (scaffold*[Title/Abstract] OR composit*[Title/Abstract])) OR ((nanomolecule*[Title/Abstract] OR nanoparticle*[Title/Abstract]) AND (hydroxyapatite[Title/Abstract])) (n=4,099)

n - number of hits, WoS - Web of Science Core Collection, ProQuest™ - ProQuest™ Dissertations & Theses Citation Index, KCI - Korean Citation Index, SciELO - SciELO Citation Index, CENTRAL - Cochrane Central Register of Controlled Trials, TS - Topic (article title, abstract and keywords)
