# Supplementary material for: The Impact of Nano-Hydroxyapatite Scaffold Enrichment on Bone Regeneration In Vivo—A Systematic Review
Source: Biomimetics (Basel). 2024 Jun 25;9(7):386. doi: 10.3390/biomimetics9070386 (PMC11274561; doi:10.3390/biomimetics9070386)
Supplement: Supplementary file 1 [file biomimetics-09-00386-s001.zip › Supplementary Table S2.pdf]

Supplementary Table S2. Excluded studies and reasons

No.	Study	Reason
1	Ahmadipour S, Varshosaz J, Hashemibeni B, Manshaei M, Safaeian L. In vivo assessment of bone repair by an injectable nanocomposite scaffold for local co-delivery of autologous platelet-rich plasma and calcitonin in a rat model. Drug Dev Ind Pharm. 2022;48(3):98–108.	A
2	Bahraminasab M, Doostmohammadi N, Talebi A, Arab S, Alizadeh A, Ghanbari A, et al. 3D printed polylactic acid/gelatin-nano-hydroxyapatite/platelet-rich plasma scaffold for critical-sized skull defect regeneration. Biomed Eng Online. 2022;21(1):86.	A
3	Chen J, Qian Y, Li H, Zuo W, Sun W, Xing D, et al. Lysophosphatidic Acid/Polydopamine-Modified nHA Composite Scaffolds for Enhanced Osteogenesis via Upregulating the Wnt/Beta-Catenin Pathway. ACS Appl Mater Interfaces. 2024;16(11):13466–80.	A
4	Deng N, Sun J, Li Y, Chen L, Chen C, Wu Y, et al. Experimental study of rhBMP-2 chitosan nano-sustained release carrier-loaded PLGA/nHA scaffolds to construct mandibular tissue-engineered bone. Arch Oral Biol. 2019;102:16–25.	A
5	Geng Z, Wang X, Zhao J, Li Z, Ma L, Zhu S, et al. The synergistic effect of strontium-substituted hydroxyapatite and microRNA-21 on improving bone remodeling and osseointegration. Biomater Sci. 2018;6(10):2694–703.	A
6	Hachinohe Y, Taira M, Hoshi M, Yoshida D, Hatakeyama W, Sawada T, et al. Self-Prepared Hyaluronic Acid/Alkaline Gelatin Composite with Nano-Hydroxyapatite and Bone Morphogenetic Protein for Cranial Bone Formation. Int J Mol Sci [Internet]. 2023 [cited 1AD Jan 1];24(2).	A
7	Jensen T, Baas J, Dolathshahi-Pirouz A, Jacobsen T, Singh G, Nygaard JV, et al. Osteopontin functionalization of hydroxyapatite nanoparticles in a PDLLA matrix promotes bone formation. J Biomed Mater Res A. 2011;99(1):94–101.	A
8	Ji Y, Wang M, Liu W, Chen C, Cui W, Sun T, et al. Chitosan/nHAC/PLGA microsphere vehicle for sustained release of rhBMP-2 and its derived synthetic oligopeptide for bone regeneration. J Biomed Mater Res A. 2017;105(6):1593–606.	A
9	Jiang Y, Pan X, Yao M, Han L, Zhang X, Jia Z, et al. Bioinspired adhesive and tumor microenvironment responsive nanoMOFs assembled 3D-printed scaffold for anti-tumor therapy and bone regeneration. Nano Today [Internet]. 2021;39.	A
10	Kamali A, Oryan A, Hosseini S, Ghanian MH, Alizadeh M, Baghaban Eslaminejad M, et al. Cannabidiol-loaded microspheres incorporated into osteoconductive scaffold enhance mesenchymal stem cell recruitment and regeneration of critical-sized bone defects. Mater Sci Eng C Mater Biol Appl. 2019;101:64–75.	A
11	Kang Y, Xu J, Meng L, Su Y, Fang H, Liu J, et al. 3D bioprinting of dECM/Gel/QCS/nHAp hybrid scaffolds laden with mesenchymal stem cell-derived exosomes to improve angiogenesis and osteogenesis. Biofabrication [Internet]. 2023 [cited 2AD Jan 1];15(2).	A
12	Li J, Xu Q, Teng B, Yu C, Li J, Song L, et al. Investigation of angiogenesis in bioactive 3-dimensional poly(d,l-lactide-co-glycolide)/nano-hydroxyapatite scaffolds by in vivo multiphoton microscopy in murine calvarial critical bone defect. Acta Biomater. 2016;42:389–99.	A
13	Li X, Yin HM, Luo E, Zhu S, Wang P, Zhang Z, et al. Accelerating Bone Healing by Decorating BMP-2 on Porous Composite Scaffolds. ACS Appl Bio Mater. 2019;2(12):5717–26.	A

14	Liu J, Mao K, Liu Z, Wang X, Cui F, Guo W, et al. Injectable biocomposites for bone healing in rabbit femoral condyle defects. PLoS One. 2013;8(10):e75668.	A
15	Liu X, Zhao K, Gong T, Song J, Bao C, Luo E, et al. Delivery of growth factors using a smart porous nanocomposite scaffold to repair a mandibular bone defect. Biomacromolecules. 2014;15(3):1019–30.	A
16	Lu W, Zeng M, Liu W, Ma T, Fan X, Li H, et al. Human urine-derived stem cell exosomes delivered via injectable GelMA templated hydrogel accelerate bone regeneration. Mater Today Bio. 2023;19:100569.	A
17	Qiu Y, Xu X, Guo W, Zhao Y, Su J, Chen J. Mesoporous Hydroxyapatite Nanoparticles Mediate the Release and Bioactivity of BMP-2 for Enhanced Bone Regeneration. ACS Biomater Sci Eng. 2020;6(4):2323–35.	A
18	Salehi M, Bastami F, Rezai Rad M, Nokhbatolfoghahaei H, Paknejad Z, Nazeman P, et al. Investigation of cell-free poly lactic acid/nanoclay scaffolds prepared via thermally induced phase separation technique containing hydroxyapatite nanocarriers of erythropoietin for bone tissue engineering applications. Polymers for Advanced Technologies. 2021;32(2):670–80.	A
19	Shen X, Zhang Y, Gu Y, Xu Y, Liu Y, Li B, et al. Sequential and sustained release of SDF-1 and BMP-2 from silk fibroin-nanohydroxyapatite scaffold for the enhancement of bone regeneration. Biomaterials. 2016;106:205–16.	A
20	Sun J, Zhang Y, Li B, Gu Y, Chen L. Controlled release of BMP-2 from a collagen-mimetic peptide-modified silk fibroin-nanohydroxyapatite scaffold for bone regeneration. J Mater Chem B. 2017;5(44):8770–9.	A
21	Sun T, Qu Y, Cui W, Yang L, Ji Y, Yu W, et al. Evaluation of osteogenic inductivity of a novel BMP2-mimicking peptide P28 and P28-containing bone composite. J Biomed Mater Res A. 2018;106(1):210–20.	A
22	Sun X, Mao Y, Liu B, Gu K, Liu H, Du W, et al. Mesenchymal Stem Cell-Derived Exosomes Enhance 3D-Printed Scaffold Functions and Promote Alveolar Bone Defect Repair by Enhancing Angiogenesis. Journal of Personalized Medicine [Internet]. 2023;13(2).	A
23	Tan R, She Z, Wang M, Yu X, Jin H, Feng Q. Repair of rat calvarial bone defects by controlled release of rhBMP-2 from an injectable bone regeneration composite. J Tissue Eng Regen Med. 2012;6(8):614–21.	A
24	Wang B, Yuan S, Xin W, Chen Y, Fu Q, Li L, et al. Synergic adhesive chemistry-based fabrication of BMP-2 immobilized silk fibroin hydrogel functionalized with hybrid nanomaterial to augment osteogenic differentiation of rBMSCs for bone defect repair. Int J Biol Macromol. 2021;192:407–16.	A
25	Wang G, Zhu Y, Zan X, Li M. Endowing Orthopedic Implants' Antibacterial, Antioxidation, and Osteogenesis Properties Through a Composite Coating of Nano-Hydroxyapatite, Tannic Acid, and Lysozyme. Front Bioeng Biotechnol. 2021;9:718255.	A
26	Wang Q, Zhang Y, Li B, Chen L. Controlled dual delivery of low doses of BMP-2 and VEGF in a silk fibroin-nanohydroxyapatite scaffold for vascularized bone regeneration. J Mater Chem B. 2017;5(33):6963–72.	A

27	Ye K, Liu D, Kuang H, Cai J, Chen W, Sun B, et al. Three-dimensional electrospun nanofibrous scaffolds displaying bone morphogenetic protein-2-derived peptides for the promotion of osteogenic differentiation of stem cells and bone regeneration. J Colloid Interface Sci. 2019;534:625–36.	A
28	Zhang L, Dong Y, Liu Y, Liu X, Wang Z, Wan J, et al. Multifunctional hydrogel/platelet-rich fibrin/nanofibers scaffolds with cell barrier and osteogenesis for guided tissue regeneration/guided bone regeneration applications. Int J Biol Macromol. 2023;253:126960.	A
29	Zhu Y, Cao N, Zhang Y, Cao G, Hao C, Liu K, et al. The Ability and Mechanism of nHAC/CGF in Promoting Osteogenesis and Repairing Mandibular Defects. Nanomaterials (Basel) [Internet]. 2022 [cited 1AD Jan 1];12(2).	A
30	Biazar E, Heidari Keshel S. Electrospun poly (3-hydroxybutyrate-co-3-hydroxyvalerate)/hydroxyapatite scaffold with unrestricted somatic stem cells for bone regeneration. ASAIO J. 2015;61(3):357–65.	B
31	Cai Y, Tan X, Zhao L, Zhang R, Zhu T, Du Y, et al. Synthesis of a novel bFGF/nHAP/COL bone tissue engineering scaffold for mandibular defect regeneration in a rabbit model. Journal of Hard Tissue Biology. 2018;27(1):85–94.	B
32	Ding Q, Qu Y, Shi K, He X, Chen Z, Yang Y, et al. Preparation of Bone Marrow Mesenchymal Stem Cells Combined with Hydroxyapatite/Poly(d,l-lactide) Porous Microspheres for Bone Regeneration in Calvarial Defects. ACS Appl Bio Mater. 2018;1(4):1084–93.	B
33	Emamgholi A, Rahimi M, Kaka G, Sadraie SH, Najafi S. Presentation of a novel model of chitosan- polyethylene oxide-nanohydroxyapatite nanofibers together with bone marrow stromal cells to repair and improve minor bone defects. Iran J Basic Med Sci. 2015;18(9):887–93.	B
34	Hu J, Yang Z, Zhou Y, Liu Y, Li K, Lu H. Porous biphasic calcium phosphate ceramics coated with nano-hydroxyapatite and seeded with mesenchymal stem cells for reconstruction of radius segmental defects in rabbits. J Mater Sci Mater Med. 2015;26(11):257.	B
35	Ji X, Yuan X, Ma L, Bi B, Zhu H, Lei Z, et al. Mesenchymal stem cell-loaded thermosensitive hydroxypropyl chitin hydrogel combined with a three-dimensional-printed poly(ε-caprolactone) /nano-hydroxyapatite scaffold to repair bone defects via osteogenesis, angiogenesis and immunomodulation. Theranostics. 2020;10(2):725–40.	B
36	Li X, Zhang R, Tan X, Li B, Liu Y, Wang X. Synthesis and Evaluation of BMMSC-seeded BMP-6/nHAG/GMS Scaffolds for Bone Regeneration. Int J Med Sci. 2019;16(7):1007–17.	B
37	Liu H, Peng H, Wu Y, Zhang C, Cai Y, Xu G, et al. The promotion of bone regeneration by nanofibrous hydroxyapatite/chitosan scaffolds by effects on integrin-BMP/Smad signaling pathway in BMSCs. Biomaterials. 2013;34(18):4404–17.	B
38	Liu Q, Feng L, Chen Z, Lan Y, Liu Y, Li D, et al. Ultrasmall Superparamagnetic Iron Oxide Labeled Silk Fibroin/Hydroxyapatite Multifunctional Scaffold Loaded With Bone Marrow-Derived Mesenchymal Stem Cells for Bone Regeneration. Front Bioeng Biotechnol. 2020;8:697.	B
39	Machado-Paula MM, Corat MAF, de Vasconcellos LMR, Araújo JCR, Mi G, Ghannadian P, et al. Rotary Jet-Spun Polycaprolactone/Hydroxyapatite and Carbon Nanotube Scaffolds Seeded with Bone Marrow Mesenchymal Stem Cells Increase Bone Neoformation. ACS Appl Bio Mater. 2022;5(3):1013–24.	B

40	Ruan SQ, Yan L, Deng J, Huang WL, Jiang DM. Preparation of a biphase composite scaffold and its application in tissue engineering for femoral osteochondral defects in rabbits. Int Orthop. 2017;41(9):1899–908.	B
41	Shi Z, Zhong Q, Chen Y, Gao J, Pan X, Lian Q, et al. Nanohydroxyapatite, Nanosilicate-Reinforced Injectable, and Biomimetic Gelatin-Methacryloyl Hydrogel for Bone Tissue Engineering. Int J Nanomedicine. 2021;16:5603–19.	B
42	Song W, Zhao L, Gao Y, Han C, Gao S, Guo M, et al. Dual growth factor-modified microspheres nesting human-derived umbilical cord mesenchymal stem cells for bone regeneration. J Biol Eng. 2023;17(1):43.	B
43	Stamnitz S, Krawczenko A, Szałaj U, Górecka Ż, Antończyk A, Kiełbowicz Z, et al. Osteogenic Potential of Sheep Mesenchymal Stem Cells Preconditioned with BMP-2 and FGF-2 and Seeded on an nHAP-Coated PCL/HAP/β-TCP Scaffold. Cells [Internet]. 2022 [cited 10AD Jan 1];11(21).	B
44	Su J, Xu H, Sun J, Gong X, Zhao H. Dual delivery of BMP-2 and bFGF from a new nano-composite scaffold, loaded with vascular stents for large-size mandibular defect regeneration. Int J Mol Sci. 2013;14(6):12714–28.	B
45	Zhang P, Wu H, Wu H, Lù Z, Deng C, Hong Z, et al. RGD-conjugated copolymer incorporated into composite of poly(lactide-co-glycotide) and poly(L-lactide)-grafted nanohydroxyapatite for bone tissue engineering. Biomacromolecules. 2011;12(7):2667–80.	B
46	Zhu S, Zhang X, Chen X, Wang Y, Li S, Qiu G, et al. Nano-Hydroxyapatite Scaffold Based on Recombinant Human Bone Morphogenetic Protein 2 and Its Application in Bone Defect Repair. J Biomed Nanotechnol. 2021;17(7):1330–8.	B
47	Chen S, Cheng D, Bao W, Ding R, Shen Z, Huang W, et al. Polydopamine-Functionalized Strontium Alginate/Hydroxyapatite Composite Microhydrogel Loaded with Vascular Endothelial Growth Factor Promotes Bone Formation and Angiogenesis. ACS Appl Mater Interfaces. 2024;16(4):4462–77.	C
48	Chen T, Gomez AW, Zuo Y, Li X, Zhang Z, Li Y, et al. Osteogenic potential and synergistic effects of growth factors delivered from a bionic composite system. J Biomed Mater Res A. 2016;104(3):659–68.	C
49	Cheng W, Ding Z, Zheng X, Lu Q, Kong X, Zhou X, et al. Injectable hydrogel systems with multiple biophysical and biochemical cues for bone regeneration. Biomater Sci. 2020;8(9):2537–48.	C
50	Curtin CM, Tierney EG, McSorley K, Cryan SA, Duffy GP, O'Brien FJ. Combinatorial gene therapy accelerates bone regeneration: non-viral dual delivery of VEGF and BMP2 in a collagen-nanohydroxyapatite scaffold. Adv Healthc Mater. 2015;4(2):223–7.	C
51	Ji J, Tong X, Huang X, Zhang J, Qin H, Hu Q. Patient-Derived Human Induced Pluripotent Stem Cells From Gingival Fibroblasts Composited With Defined Nanohydroxyapatite/Chitosan/Gelatin Porous Scaffolds as Potential Bone Graft Substitutes. Stem Cells Transl Med. 2016;5(1):95–105.	C
52	Jiang SJ, Wang MH, Wang ZY, Gao HL, Chen SM, Cong YH, et al. Radially Porous Nanocomposite Scaffolds with Enhanced Capability for Guiding Bone Regeneration In Vivo. Advanced Functional Materials [Internet]. 2022;32(18).	C

53	Li X, Ye F, Li G, Cui J, Liu Y, Yang L, et al. 3D Printed Hydroxyapatite/Silk Fibroin/Polycaprolactone Artificial Bone Scaffold and Bone Tissue Engineering Materials Constructed with Double-Transfected Bone Morphogenetic Protein-2 and Vascular Endothelial Growth Factor Mesenchymal Stem Cells to Repair Rabbit Radial Bone Defects. NANOSCIENCE AND NANOTECHNOLOGY LETTERS. 2020;12(3):368–75.	C
54	Skelly JD, Lange J, Filion TM, Li X, Ayers DC, Song J. Vancomycin-bearing synthetic bone graft delivers rhBMP-2 and promotes healing of critical rat femoral segmental defects. Clin Orthop Relat Res. 2014;472(12):4015–23.	C
55	Wang Q, Xia Q, Wu Y, Zhang X, Wen F, Chen X, et al. 3D-Printed Atsttrin-Incorporated Alginate/Hydroxyapatite Scaffold Promotes Bone Defect Regeneration with TNF/TNFR Signaling Involvement. Adv Healthc Mater. 2015;4(11):1701–8.	C
56	Wang Z, Xu Y, Wang Y, Ito Y, Zhang P, Chen X. Enhanced in Vitro Mineralization and in Vivo Osteogenesis of Composite Scaffolds through Controlled Surface Grafting of L-Lactic Acid Oligomer on Nanohydroxyapatite. Biomacromolecules. 2016;17(3):818–29.	C
57	Yan S, Feng L, Zhu Q, Yang W, Lan Y, Li D, et al. Controlled Release of BMP-2 from a Heparin-Conjugated Strontium-Substituted Nanohydroxyapatite/Silk Fibroin Scaffold for Bone Regeneration. ACS Biomater Sci Eng. 2018;4(9):3291–303.	C
58	Canciani E, Straticò P, Varasano V, Dellavia C, Sciarrini C, Petrizzi L, et al. Polylevolysine and Fibronectin-Loaded Nano-Hydroxyapatite/PGLA/Dextran-Based Scaffolds for Improving Bone Regeneration: A Histomorphometric in Animal Study. Int J Mol Sci [Internet]. 2023 [cited 5AD Jan 1];24(9).	D
59	Kim SS, Gwak SJ, Kim BS. Orthotopic bone formation by implantation of apatite-coated poly(lactide-co-glycolide)/hydroxyapatite composite particulates and bone morphogenetic protein-2. J Biomed Mater Res A. 2008;87(1):245–53.	D
60	Liao SS, Cui FZ, Zhang W, Feng QL. Hierarchically biomimetic bone scaffold materials: nano-HA/collagen/PLA composite. J Biomed Mater Res B Appl Biomater. 2004;69(2):158–65.	D
61	Martínez-Sanz E, Varghese OP, Kisiel M, Engstrand T, Reich KM, Bohner M, et al. Minimally invasive mandibular bone augmentation using injectable hydrogels. J Tissue Eng Regen Med. 2012;6:s15-23.	D
62	Osathanon T, Linnes ML, Rajachar RM, Ratner BD, Somerman MJ, Giachelli CM. Microporous nanofibrous fibrin-based scaffolds for bone tissue engineering. Biomaterials. 2008;29(30):4091–9.	D
63	Wang T, Guo S, Zhang H, Chen Y, Cai Y. Injectable hydrogel delivering bone morphogenetic protein-2, vascular endothelial growth factor, and adipose-derived stem cells for vascularized bone tissue engineering. Journal of Drug Delivery Science and Technology [Internet]. 2020;57.	D
64	Wang X, Wu X, Xing H, Zhang G, Shi Q, E L, et al. Porous Nanohydroxyapatite/Collagen Scaffolds Loading Insulin PLGA Particles for Restoration of Critical Size Bone Defect. ACS Appl Mater Interfaces. 2017;9(13):11380–91.	D
65	Zhang Z, Wu G, Cao Y, Liu C, Jin Y, Wang Y, et al. Self-assembling peptide and nHA/CTS composite scaffolds promote bone regeneration through increasing seed cell adhesion. Mater Sci Eng C Mater Biol Appl. 2018;93:445–54.	D
66	Zhao W, Li Y, Zhou A, Chen X, Li K, Chen S, et al. Controlled release of basic fibroblast growth factor from a peptide biomaterial for bone regeneration. R Soc Open Sci. 2020;7(4):191830.	D

67	Abe T, Sumi K, Kunimatsu R, Oki N, Tsuka Y, Awada T, et al. Bone Regeneration in a Canine Model of Artificial Jaw Cleft Using Bone Marrow-Derived Mesenchymal Stem Cells and Carbonate Hydroxyapatite Carrier. Cleft Palate Craniofac J. 2020;57(2):208–17.	E
68	Fang CH, Lin YW, Lin FH, Sun JS, Chao YH, Lin HY, et al. Biomimetic Synthesis of Nanocrystalline Hydroxyapatite Composites: Therapeutic Potential and Effects on Bone Regeneration. Int J Mol Sci [Internet]. 2019 [cited 11AD Jan 1];20(23).	E
69	Kim BS, Yang SS, Kim CS. Incorporation of BMP-2 nanoparticles on the surface of a 3D-printed hydroxyapatite scaffold using an ε-polycaprolactone polymer emulsion coating method for bone tissue engineering. Colloids Surf B Biointerfaces. 2018;170:421–9.	E
70	Limlawan P, Insin N, Marger L, Freudenreich M, Durual S, Vacharaksa A. 3D-printed TCP-HA scaffolds delivering MicroRNA-302a-3p improve bone regeneration in a mouse calvarial model. BDJ Open. 2023;9(1):50.	E
71	Chen XM, Ma X, Shao NQ, Dai YX, Gao Q, Fu JF. Nano-hydroxyapatite/silk fibroin composite materials loaded with recombinant human bone morphogenetic protein 2 used for spinal fusion. Chinese Journal of Tissue Engineering Research. 2017;21(18):2802–7.	F
72	Du GQ, Sun J, Li YL, Chen LQ, Chen C, Deng N, et al. Reconstruction of mandibular defects with tissue-engineered bone using 3d bionic printing technology. Chinese Journal of Tissue Engineering Research. 2018;22(18):2813–9.	F
73	Hu B, Yang SH, Xiao DM, Wang DP, Guan H, Yang XJ. Repairing bone deffects with vascular endothelial growth factor/nano-hydroxyapatite composite artificial bone. Journal of Clinical Rehabilitative Tissue Engineering Research. 2008;12(32):6209–12.	F
74	Lu XJ, Zhao YZ, Wei W, Ting Z, Chen HJ, Wei Z, et al. Recombinant adenovirus-mediated bone morphogenetic protein 9 versus bone morphogenetic protein 2 combined with nano-hydroxyapatite/polyamide composites in repair of radial defects. Journal of Clinical Rehabilitative Tissue Engineering Research. 2010;14(21):3815–8.	F
75	Wan Z, Tie C. Effect of Recombinant Human Growth Hormon Combined with Nano-Hydroxyapatiteon on Tibia Defect Repair of Rabbits. Medical Journal of Wuhan University. 2017;38(3):366–70.	F
76	Wu DJ, Hao AH, Zhang C, Cui FZ, Wang XW, Gao CZ, et al. Promoting of angiogenesis and osteogenesis in radial critical bone defect regions of rabbits with nano-hydroxyapatite/collagen/PLA scaffolds plus endothelial progenitor cells. National Medical Journal of China. 2012;92(23):1630–4.	F
77	Wu GX, Wang JG, Du XY. Bone marrow mononuclear cells combined with nano-hydroxyapatite/collagen for repair of mandibular defects. Chinese Journal of Tissue Engineering Research. 2013;17(38):6791–6.	F
78	Zang XL, Sun J, Li YL, Chen LQ, Yang XC, Liang LQ, et al. 3D-bioprinting manufacturing polylactic-co-glycolic acid/nano-hydroxyapatite scaffold/bone morphogenetic protein-2 sustained release composite. Chinese Journal of Tissue Engineering Research. 2016;20(16):2405–11.	F
79	Zhang X, Zhang Y, Cui FZ. Bioactivity of the peptide P17-bone morphogenetic protein-2/nano-hydroxyapatite-mineralized collagen composite. Chinese Journal of Tissue Engineering Research. 2020;24(22):3547–52.	F
80	He Y, Dong Y, Cui F, Chen X, Lin R. Ectopic osteogenesis and scaffold biodegradation of nano-hydroxyapatite-chitosan in a rat model. PLoS One. 2015;10(8):e0135366.	G

81	Li X, Zhang R, Li B, Tan X, Wang X. Biocompatible nHA/Col-BMP-9/GM scaffold: Synthesis, characterization, and effects on bone marrow mesenchymal stem cells. Journal of Hard Tissue Biology. 2019;28(2):175–84.	G
82	Salgado CL, Teixeira BIB, Monteiro FJM. Biomimetic Composite Scaffold With Phosphoserine Signaling for Bone Tissue Engineering Application. Front Bioeng Biotechnol. 2019;7:206.	G
83	Shaikh S, Gupta S, Mishra A, Sheikh PA, Singh P, Kumar A. Laser-assisted synthesis of nano-hydroxyapatite and functionalization with bone active molecules for bone regeneration. Colloids Surf B Biointerfaces. 2024;237:113859.	G
84	Tenkumo T, Vanegas Sáenz JR, Nakamura K, Shimizu Y, Sokolova V, Epple M, et al. Prolonged release of bone morphogenetic protein-2 in vivo by gene transfection with DNA-functionalized calcium phosphate nanoparticle-loaded collagen scaffolds. Mater Sci Eng C Mater Biol Appl. 2018;92:172–83.	G
85	Zhang C, Hu YY, Cui FZ, Zhang SM, Ruan DK. A study on a tissue-engineered bone using rhBMP-2 induced periosteal cells with a porous nano-hydroxyapatite/collagen/poly(L-lactic acid) scaffold. Biomed Mater. 2006;1(2):56–62.	G
86	Bal Z, Korkusuz F, Ishiguro H, Okada R, Kushioka J, Chijimatsu R, et al. A novel nano-hydroxyapatite/synthetic polymer/bone morphogenetic protein-2 composite for efficient bone regeneration. Spine J. 2021;21(5):865–73.	H
87	Shiels S, Oh S, Bae C, Guda T, Singleton B, Dean DD, et al. Evaluation of BMP-2 tethered polyelectrolyte coatings on hydroxyapatite scaffolds in vivo. J Biomed Mater Res B Appl Biomater. 2012;100(7):1782–91.	H
88	Wang L, Zheng L, Li C, Dong S, A L, Zhou Y. Adrenomedullin delivery in microsphere-scaffold composite for remodeling of the alveolar bone following tooth extraction: An experimental study in the rat. BioMedical Engineering Online [Internet]. 2013;12(1).	H
89	Zhu W, Wang D, Zhang X, Lu W, Han Y, Ou Y, et al. Experimental study of nano-hydroxyapatite/recombinant human bone morphogenetic protein-2 composite artificial bone. Artif Cells Blood Substit Immobil Biotechnol. 2010;38(3):150–6.	H
90	Du B, Gao Y, Deng Y, Zhao Y, Lai C, Guo Z, et al. Local delivery of rhVEGF165 through biocoated nHA/coral block grafts in critical-sized dog mandible defects: a histological study at the early stages of bone healing. Int J Clin Exp Med. 2015;8(4):4940–53.	I
91	Kuttappan S, Jo JI, Menon D, Ishimoto T, Nakano T, Nair SV, et al. ONO-1301 loaded nanocomposite scaffolds modulate cAMP mediated signaling and induce new bone formation in critical sized bone defect. Biomater Sci. 2020;8(3):884–96.	I
92	Xie LN, Wang P, Pan JL, Sun Z, Cui FZ. The effect of platelet-rich plasma with mineralized collagen-based scaffold on mandible defect repair in rabbits. Journal of Bioactive and Compatible Polymers. 2010;25(6):603–21.	I
93	Niu X, Fan Y, Liu X, Li X, Li P, Wang J, et al. Repair of bone defect in femoral condyle using microencapsulated chitosan, nanohydroxyapatite/collagen and poly(L-lactide)-based microsphere-scaffold delivery system. Artif Organs. 2011;35(7):E119-28.	J
94	Rivero G, Aldana AA, Frontini Lopez YR, Liverani L, Boccacini AR, Bustos DM, et al. 14-3-3ε protein-immobilized PCL-HA electrospun scaffolds with enhanced osteogenicity. J Mater Sci Mater Med. 2019;30(9):99.	K

95	Liu B, Chen P, Wang ZY, Ke J, Li XH, Wang ZW. Nano-hydroxyapatite/collagen composited with recombinant human bone morphogenetic protein-2 and titanium membrane in repairing peripheral bone defects of instant dental implants. Journal of Clinical Rehabilitative Tissue Engineering Research. 2009;13(29):5779–83.	L

A - Composite scaffold with less than 30% of nHAp, or unknown % of nHAp (n=29); B - Cells loaded on scaffold (n=17); C - No group of bioactive molecules alone as addition to basic scaffold (n=11); D - Absence of adequate control group (n=9); E - Use of HAp that is not nano-size (n=4); F- Written in Chinese language (n=9); G - Ectopic bone formation model (n=6); H - Non-critical size bone defect (n=4); I - Less than 4 animals per each test group (n=3); J - Unclear sample size (n=1); K - *in vitro* study (n=1); L - not retrieved in full-text (n = 1).
